# In Memoriam

**DOI:** 10.21307/jofnem-2021-097

**Published:** 2021-11-06

**Authors:** James G. Baldwin, Jonathan D. Eisenback

**Affiliations:** 1 Department of Nematology, University of California, Riverside 92521; 2 Department of Plant Pathology, Virginia Tech University, Blacksburg 24061

**Hedwig Hirschmann Triantaphyllou (1927-2021)**

**Figure:**
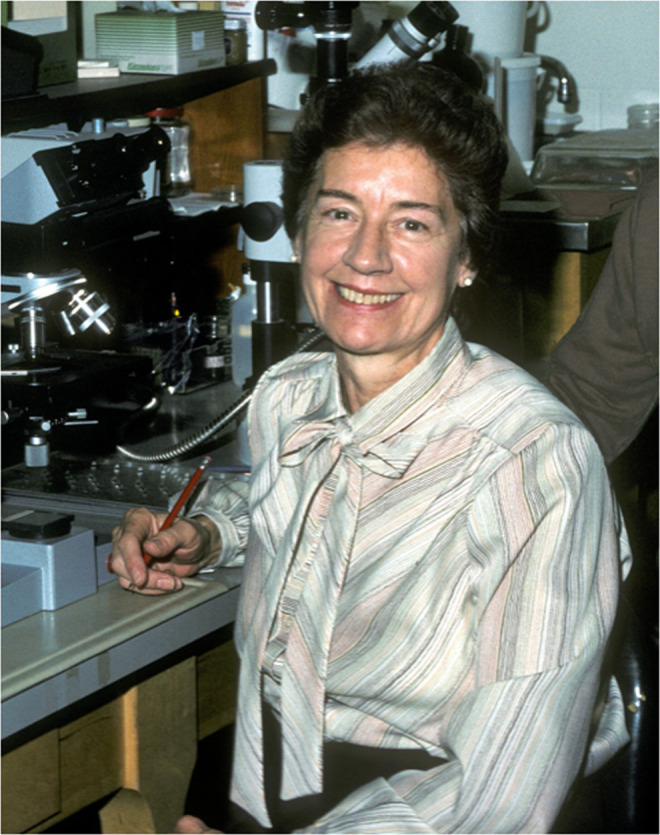


Hedwig Hirschmann Triantaphyllou passed away on September 22, 2021. She was born in Fürth Germany on January 16, 1927, the daughter of the late Frederick and Ferdinandine Hirschmann. From an early age Hedwig was interested in science and she shared a love for music with her musician/conductor father. Her father’s death at age 54 in 1950 put responsibility on young Hedwig toward supporting herself and her mother.

Hedwig completed her Bachelor of Science and Ph. D. degrees at the University of Erlangen in 1951 under the mentorship of Professor Hans-Jürgen Stammer and there she continued as a postdoctorate until 1954. Early in this period the university facilities were badly impacted by war such that sixty graduate students in zoology, including Hedwig, shared a single light microscope that was scheduled 24 hours a day and 7 days a week. Nevertheless, even under these circumstances, Hedwig made important contributions on taxonomy, biology and community structure of free-living nematodes. In the Stammer lab, the care and precision of her early taxonomic observations, together with those of her classmate, Günther Osche, included building understanding of rhabditids and diplogasterids. It could not be envisioned at the time that this work, including Hedwig’s description of mouth dimorphism within a single diplogasterid species, would contribute to the foundation for basic biology model systems including *Caenorhabditis* and *Pristionchus*.

Early in the 1950s, as interest grew in nematodes of agricultural importance, Dr. Hirschmann met Gotthold Steiner, nematologist of the United States Department of Agriculture. Steiner encouraged her to move to the United States noting an available position at North Carolina State University (NCSU). She was hired at NCSU 1954 and advanced to full Professor in 1967.

At NCSU, through the power of superb insight, meticulous techniques, broad understanding of anatomy and morphology, as well as careful observation of large samples, Professor Hirschmann unraveled difficult problems of species diagnosis and interspecific variability. Her exacting work set the standard for such descriptions while impacting understanding of agriculturally significant genera including *Meloidogyne*, *Heterodera, Anguina, Ditylenchus, Radopholus* and *Belonolaimus*. Professor Hirschmann brought the same meticulous approach to elucidating details of reproductive biology, life history and some of the first work on embryogenesis and postembryogenesis of plant parasitic Tylenchida, including species of *Ditylenchus*, *Meloidogyne, Heterodera, Anguina, Helicotylenchus, Pratylenchus* and *Meloidodera*. Investigations of developmental biology included elucidating diversity among species in modes of reproduction and sex determination and, in collaboration with her husband, work on cytogenetics that formed the basis for insightful hypotheses of evolution and speciation among plant parasitic nematodes. Later in her career Professor Hirschmann’s contributions to understanding morphology and development were complemented by new levels of precision using transmission and scanning electron microscopy.

The care of Professor Hirschmann’s publications, including exacting, unambiguous illustrations, resulted in invitations to write review articles and book chapters on nematode morphology, reproduction, and systematics. Particularly impactful was her role in the *International Meloidogyne Project* (IMP) from 1975–1985 where she contributed to a number of books, practical guides to identification, classification, and taxonomic principles that continue to be a primary resource throughout the world. In the context of IMP, she was involved in training and collaborating with many scientists from the participating 70 developing countries. In conjunction with IMP Professor Hirschmann confirmed the identity and maintained frozen stocks of about 80 isolates of *Meloidogyne* from throughout the world; this collection is since curated at Clemson University.

Professor Hirschmann excelled as a classroom teacher as well as a graduate study supervisor, instilling in her students an absolute high standard for their work. Her course in nematode morphology and taxonomy, taught for more than 30 years, served as a model for clarity, concern for accuracy, technical precision and detail for graduate lectures and laboratories. Many of her students from that class now teach their own classes. These former students continue to build upon the framework from her class, and thereby extend her teaching to new generations of nematologists. Through Professor Hirschmann’s effective personal style of mentoring she will always be highly respected and beloved by her five Ph. D. students. The special mark of Professor Hirschmann was consistent encouragement; yet, she never hesitated to question, challenge and motivate her students to the same high standard she held for herself. Clearly, her concerns and even demands for detail and accuracy contributed much to the education and success of all students mentored by her and those who enrolled in her challenging graduate course in nematology.

Professor Hirschmann’s contributions have been recognized by the 1962 Sigma Xi Research Award, 1975 Outstanding Educator Award from North Carolina State University, the coveted Ruth Allen Award (1993) by The American Phytopathological Society, and 1981 Fellow of the Society of Nematologists. She also received a special Susan B. Anthony recognition for her pioneering research as a “woman scientist.” Thus, Professor Hirschmann, through her research and graduate education programs, has had a profound impact on the sciences, particularly of nematology and plant pathology.

Although Professor Hirschmann retired from the NCSU Department of Plant Pathology in 1992, her research on morphology and taxonomy of the root-knot nematodes continued. During the period of 1951 through 1999, her research, publications, and teaching contributed greatly to rapid advancement of the young science of nematology. She influenced many, not only by her scientific contributions but by her kindness, graciousness and generosity. Together with her husband, in 1999 she generously created endowments in support of NCSU graduate students in programs in Plant Pathology and Genetics as well as for the NCSU library collections in those disciplines.

Dr. Hirschmann was married for 49 years to the late Dr. Anastasios C. “Tasso” Triantaphyllou, Professor of the NCSU Department of Genetics. Beyond their research collaboration, they entertained friends with Hedwig’s mastery of the piano and Tasso’s talent on the mandolin. What began as a duet quickly advanced to a small band including a second mandolin and two guitars. Hedwig and Tasso enjoyed weekend sailing at North Carolina lakes. However, this hobby grew increasingly competitive and the skilled sailing team advanced nationally; in 1968 they finished in third place in the President Cup Races on the Potomac River in Washington DC.

Dr. Hedwig Hirschmann and Dr. Tasso Triantaphyllou are survived by their son Chris. In her work, Hedwig touched many lives with her contributions and commitment to the excellence, clarity and attention to detail that was her hallmark. Beyond professional contributions she influenced many with her friendship, encouragement, kindness and generosity. Hedwig will be greatly missed.

